# Real-World Data on the Effectiveness and Safety of wilate for the Treatment of von Willebrand Disease

**DOI:** 10.1055/s-0041-1730283

**Published:** 2021-07-04

**Authors:** Michelle Sholzberg, Kate Khair, Hassan Yaish, George Rodgers, Maria Sol Cruz, Cesar Montaño Mejía, Zuzana Čermáková, Davide Matino, Jerome Teitel, Alpha Barrie, Sylvia Werner, Mario von Depka Prondzinski

**Affiliations:** 1 Departments of Medicine and Laboratory Medicine & Pathobiology, St. Michael's Hospital, Li Ka Shing Knowledge Institute, University of Toronto, Toronto, Canada; 2 Department of Medicine, St. Michael's Hospital, University of Toronto, Toronto, Ontario, Canada; 3 Research in Children's Health, Illness and Disability (ORCHID), NIHR Biomedical Research Centre, Great Ormond Street Hospital for Children, London, United Kingdom; 4 University of Utah School of Medicine, Salt Lake City, Utah, United States; 5 Fundación de la Hemofilia de Salta, Salta, Argentina; 6 Hemolife and Universidad Tecnológica de Pereira, Pereira, Colombia; 7 Blood Center, University Hospital Ostrava, Ostrava-Poruba, Czech Republic; 8 McMaster University Medical Center, Hamilton, Ontario, Canada; 9 Octapharma USA, Inc., Paramus, New Jersey, United States; 10 Werlhof-Institut GmbH, Hannover, Germany

**Keywords:** factor VIII, observational study, prophylaxis, von Willebrand disease, von Willebrand factor

## Abstract

**Background** The efficacy and safety of wilate (human von Willebrand factor/coagulation factor VIII) in patients with von Willebrand disease (VWD) has been demonstrated in clinical trials. Here, we present real-world data on the use of wilate for the routine care of patients with VWD.

**Objectives** The objectives of this observational, prospective, phase 4 study were to evaluate the safety, tolerability, and effectiveness of wilate in on-demand treatment of bleeding episodes (BEs), long-term prophylaxis, and surgical prophylaxis among patients with any type of VWD.

**Methods** Patients were enrolled at 31 study centers in 11 countries and followed for up to 2 years. Safety endpoints included adverse drug reactions (ADRs) and drug tolerability. Effectiveness was assessed using annualized bleeding rates (ABRs) during prophylaxis and predefined criteria for the treatment of BEs and surgical prophylaxis.

**Results** A total of 111 patients (76 [68%] female) including 41 (37%) children were treated with wilate. Twenty-five patients received prophylaxis, 29 on-demand treatment, and 62 surgical prophylaxis. Tolerability was rated by patients as “excellent” for 96.2% of 6,497 infusions. No unexpected ADRs or thrombotic events were reported. Median ABR during prophylaxis was 1.9. Effectiveness was assessed as “excellent” or “good” by patients and investigators for 100% of BEs treated on-demand, 98% (patient rating) and 99% (investigator rating) of breakthrough BEs, and 99% of surgical procedures (investigator rating).

**Conclusion** wilate was safe, well tolerated, and effective for the prevention and treatment of bleeding in pediatric and adult VWD patients in a real-world setting.

## Introduction

There are three major types of von Willebrand disease (VWD), types 1 and 3 are caused by quantitative deficiency in von Willebrand factor (VWF) while a qualitative functional defect is the cause of type 2. The disease is known to be heterogeneous in genotype and phenotype. 1 Type 1 is the most common variant, accounting for 70 to 80% of cases, followed by type 2, which affects approximately 20% of patients and is subclassified into four major subtypes. 1 Type 3, the most severe form of VWD, is characterized by a near-complete absence of VWF, and affects <5% of VWD patients. 1

Treatment of VWD generally depends on the type and severity of the disease. 1 Mild-to-moderate forms of type 1, 2A, 2M, and 2N VWD may respond to treatment with desmopressin (1-deamino-8-D-arginine vasopressin, DDAVP), which is not effective in type 3 and is contraindicated in type 2B VWD. 2 In type 3 VWD, gastrointestinal and joint bleeding are particular challenges. 3 4 Several studies have demonstrated that prophylaxis is effective in patients with VWD 3 5 6 ; however, regular replacement therapy to prevent bleeding is not commonly used. 4

wilate is a plasma-derived, double virus-inactivated concentrate of freeze-dried active VWF and factor VIII (FVIII) in a physiological 1:1 ratio. Since the first approval of wilate in Germany in 2005, wilate has been approved for use in VWD and hemophilia A in a further 68 countries, with over 1.3 billion international units (IU) of wilate distributed worldwide. Several clinical trials have shown wilate to be efficacious in the prevention and treatment of bleeding, including major surgeries, in patients with all types of VWD. 6 7 8 9

Here we report the results of WIL-20, an observational, phase 4, postmarketing study designed to evaluate the safety, tolerability, and effectiveness of wilate in routine clinical practice in patients of all ages with all types of VWD across a variety of settings.

## Methods

### Study Design

WIL-20 (NCT01602419) was an observational, prospective, phase 4, postmarketing study conducted at 31 study centers in 11 countries (see acknowledgments) in accordance with the current Guideline on the Clinical Investigation of Human Plasma Derived von Willebrand Factor Products (CPMP/BPWG/220/02). 10 Relevant ethics committee approvals were obtained and written informed consent was obtained from all patients or their legal guardians. The planned observation period per patient was 2 years from study entry. The first patient was enrolled on October 25, 2010, and the last patient completed the study on December 18, 2017.

### Patients

Male and female patients of any age and with any type of inherited VWD who were prescribed wilate by their treating physicians were eligible for inclusion into the study, including patients who had been previously treated and patients who had not been previously treated.

Patients were excluded if they previously tested positive for the presence of inhibitors to VWF, had a bleeding disorder other than VWD, had a history of nonadherence to therapy, difficult venous access that would prohibit treatment, or if they were not able to follow the requirements of the study.

### Study Treatment

The choice of treatment regimen was at the discretion of the treating physician. The protocol recommended that follow-up visits take place every 3 months. Patients were asked to document any treatment-related data in their diaries, regardless of whether they were treated at home or at the treatment center. The treatment administered, type, and severity of bleeding episode (BE), and assessment of tolerability were recorded. In the case of surgical procedures, data relating to the treatments administered, blood loss, and clinical signs of bleeding were collected by the treating physician. Exposure to wilate was recorded as number of infusions and number of exposure days (EDs).

### Safety Evaluations

The primary endpoints were the incidence of recorded adverse drug reactions (ADRs) and tolerability in on-demand treatment of acute bleeding, long-term prophylaxis, and surgical prophylaxis in routine clinical practice. All ADRs occurring after initiation of study treatment were summarized by MedDRA system organ class, preferred term, intensity, and relationship (expectedness) to study treatment. ADRs were recorded in Individual Case Safety Reports (ICSRs). Tolerability of infusions for prophylaxis and for treatment of BEs was assessed by the patient using a 3-point verbal rating scale (“excellent,” “satisfactory,” or “unsatisfactory”; Supplementary Table S1 [online only]). In some instances, investigators also recorded the tolerability of infusions given for surgical prophylaxis.

Optional testing for anti-VWF antibodies was performed by enzyme-linked immunosorbent assay (ELISA) at a central laboratory (The Coagulation Laboratory, Malmö University Hospital, Sweden) after the first ED and then every 3 months during treatment. If a sample was antibody positive then the test was repeated, and if the sample was positive again, its capacity to neutralize VWF ristocetin cofactor activity (VWF:RCo) was tested using a Bethesda assay (cut-off >0.4 Bethesda unit [BU]/mL). If a sample contained >0.10 IU/mL VWF:RCo activity, an experimental assay was used to measure neutralization of VWF:CB to increase the reliability of the result. This experimental assay is a modification of the Bethesda assay that involves two control samples, one that estimates the contribution of normal plasma and one that measures the contribution from the patient plasma. The combined activity of the two controls gives the “expected” value, and a sample with a mixture of patient and normal plasma gives the “observed” value. The cut-off for positivity is an (expected − observed)/expected ratio of >0.25. If these tests were found to be negative, the anti-VWF antibody was deemed to be nonneutralizing (i.e., noninhibitory).

Thrombogenicity biomarkers including prothrombin fragments 1 + 2 (F1 + 2) and D-dimer were optionally measured by a latex-enhanced immunoturbimetric test at baseline and during follow-up. Testing was also performed in a central laboratory.

The status of immunoglobulin G and immunoglobulin M antibodies against parvovirus B19 at baseline, at each visit and at study completion was determined optionally at the discretion of the investigator via serological testing.

### Effectiveness Evaluations

Effectiveness of wilate in prophylaxis was reported as the number of BEs per year (annualized bleeding rate [ABR]). Effectiveness of wilate in the treatment of bleeding was evaluated for breakthrough BEs during prophylaxis, for BEs in patients treated on-demand, and for menstrual BEs using a 4-point hemostatic rating scale (“excellent,” “good,” “moderate,” or “none”; Supplementary Table S1 [online only]). Effectiveness in surgery was evaluated in patients who received wilate before, during, or after surgery and in whom no other VWF or FVIII concentrate was provided within 72 hours before surgery. Effectiveness of wilate in surgical procedures was also rated after surgery using a 4-point rating scale (“excellent,” “good,” “moderate,” or “none”; Supplementary Table S1 [online only]). Major surgeries were defined retrospectively using the list of surgical procedures taken from the SQLape group codes. 11 All other surgeries were considered minor.

Participants switching between prophylactic and on-demand treatment regimens were included in the analysis populations of all relevant treatment regimens for the duration of exposure to a particular regimen.

### Statistical Methods

No formal sample size calculation was performed as the study size was based on feasibility. All collected data are presented using descriptive statistics (median, mean, range, and standard deviation [SD]) using SAS, version 9.3 or higher.

## Results

### Patient Disposition

**Fig. 1:**
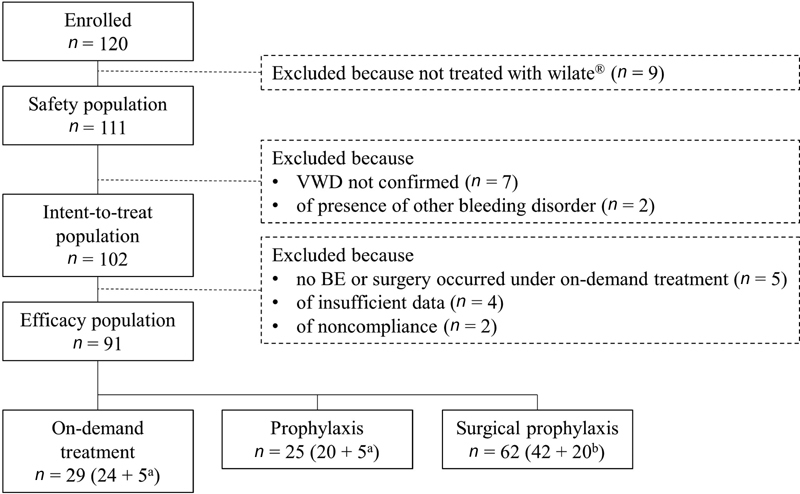
Patient disposition. ^a^ Two patients changed from on-demand treatment to prophylaxis, and three patients changed from prophylaxis to on-demand treatment. For all analyses by treatment regimen, these five patients were included in both analysis populations. Seven patients treated on-demand and two treated prophylactically also received treatment for menstrual bleeding. ^b^ Forty-two patients underwent surgery only, and 20 patients were also included in the on-demand or prophylaxis populations.

A total of 120 patients were enrolled in the study, of whom 111 received at least one treatment with wilate; 9 patients were excluded from the safety population because they did not receive any treatment with wilate ( Fig. 1 ). Of the patients who were included in the efficacy analysis, 25 received wilate prophylaxis, 29 on-demand, and 62 as surgical prophylaxis. Two patients switched from on demand to prophylaxis, and three switched from prophylaxis to on-demand; all five patients were included in both separate subpopulation analyses. In the surgical prophylaxis population, 42 patients underwent surgery only, and another 20 patients from either the prophylaxis or on-demand populations also underwent surgical intervention. Nine patients (two who were receiving prophylaxis and seven who were treated on-demand) received treatment for menstrual bleeding.

A total of 20 patients stopped study treatment prematurely, 3 of whom due to hypersensitivity (considered as ADRs). One patient experienced moderate hypersensitivity symptoms after her second infusion, another experienced mild hypersensitivity reactions on two consecutive infusions, and the third experienced moderate hypersensitivity reactions in the surgical setting. One patient stopped due to parvovirus B19 infection, likely acquired from the patient's child, and was judged as unlikely related to wilate by the investigator. Other premature discontinuations were: 3 patients stopped due to insurance-related issues, 1 patient changed to another treatment center, 1 patient's treatment center was closed, 5 patients withdrew at their own request, and 6 patients were lost to follow-up.

### Demographic and Baseline Characteristics

Approximately two-thirds of patients were females (68%) across all treatment subgroups ( Table 1 ). At baseline, the median age was 27 years (range: 0.8–83.2), with 41 (37%) of the 111 patients under 18 years of age at the time of enrollment. The majority of the patients were Caucasian ( *n* = 89; 80%), 3 patients (3%) were Asian, 1 (1%) was Black, 17 (15%) were recorded as Aboriginal, Hispanic, Turkish, Mestizo, or Lebanese, and 1 patient (1%) was of unknown ethnic origin.

**Table 1:** Baseline demographic and clinical characteristics

Parameter	Prophylaxis ( *N* = 25)	On-demand ( *N* = 29)	Surgery ( *N* = 62)	All ( *N* = 111)
Age at study entry [y], median (range)	14.0 (1.0–83.0)	20.0 (0–76.0)	35.5 (2.0–77.5)	27.0 (0.8–83.2)
Age at diagnosis of VWD [y], median (range)	7.5 (0–60.9)	15.0 (0–59.0) a	20.6 (0.4–71.4) b	13.5 (0–71.4) c
Time since diagnosis of VWD [y], median (range)	5.0 (0.2–40.0)	2.1 (0.1–60.0) a	7.0 (0–60.0) b	5.0 (−0.6–60.0) c
Sex, *n* (%)				
Male	7 (28%)	9 (31%)	19 (31%)	35 (32%)
Female	18 (72%)	20 (69%)	43 (69%)	76 (68%)
Type of VWD, *n* (%)				
1	3 (12%)	12 (41%)	35 (56%)	50 (45%)
2	8 (32%)	12 (41%)	18 (29%)	32 (29%)
2, not specified	1	3	4	5
2A	5	3	6	14
2B	1	3	4	5
2M	0	1	2	3
2N	1	2	2	5
3	14 (56%)	5 (17%)	8 (13%)	20 (18%)
Not available	0	0	1 (2%)	8 (7%)
Not applicable d	0	0	0	1 (1%)
Pre-study exposure to FVIII/VWF products by EDs, *n* (%)				
0	3 (12%)	6 (21%)	30 (48%)	37 (33%)
< 150 EDs	14 (56%)	21 (72%)	27 (44%)	57 (51%)
≥150 EDs	8 (32%)	2 (7%)	3 (5%)	14 (13%)
Not available	0	0	2 (3%)	3 (3%)

Abbreviations: ED, exposure day; FVIII, factor VIII; VWD, von Willebrand disease; VWF, von Willebrand factor.

a *N* = 28.

b *N* = 58.

c *N* = 107.

d One patient was diagnosed during the study as having hemophilia A rather than VWD.

In total, 45% of patients had type 1 VWD, 29% type 2, 18% type 3, and 7% did not have data available. One patient (1%) was later diagnosed with hemophilia A rather than VWD. Baseline levels of VWF and FVIII measured at local laboratories are provided in Supplementary Table S2 (online only). A family history of VWD was reported for 60 patients (54%). Almost all patients (99%) had no known history of VWF inhibitors; history of VWF inhibitor activity was reported in one patient. Identifiable VWF gene mutations were present in 12 patients (11%) and absent in 49 patients (44%); in the remaining 50 patients (45%), the gene mutation data were not available. Approximately one-third of patients (33%) had never been treated with a VWF/FVIII-containing product. There was a higher proportion of patients with VWD type 3 in the group receiving prophylaxis compared with the on-demand population.

### Safety and Tolerability

Throughout the observation period, 7,024 infusions were administered on 6,676 EDs. Of the 111 patients treated with wilate, 8 experienced a total of 26 ADRs, corresponding to an incidence rate of 7.2%. These were reported as eight ICSRs. The eight patients were evenly distributed across treatment regimens and age group (3 of 41 [7.3%] children and 5 of 70 [7.1%] adults). No deaths occurred in this study.

Of the 8 ICSRs, 4 were classified as mild, 3 as moderate, and 1 as severe. Three of the four mild ICSRs included an administration site reaction, injection site pain, and urticaria, which all resolved with no change to wilate therapy. The fourth mild ICSR was a mild hypersensitivity reaction that resolved, but the patient chose to withdraw from the study. All three moderate ICSRs were hypersensitivity reactions and two of these patients withdrew from the study. One patient was treated with 0.25 mg dexamethasone and 2 mg clorfenamin and continued at a diluted infusion strength (900 IU wilate diluted in 900 mL 5% dextrose). The severe ICSR was erythema infectiosum from parvovirus B19 infection. The parvovirus B19 infection was not present at baseline and had most likely been transmitted by the patient's child, who had an acute parvovirus B19 infection at the time. It was judged as unlikely related to wilate by the investigator. The patient ultimately had a spontaneous abortion and withdrew from the study; she had experienced three spontaneous abortions during the previous 2 years.

Three additional patients who were seronegative for parvovirus B19 at baseline developed parvovirus B19 antibodies at follow-up. All three patients were asymptomatic and two were negative on subsequent testing (one did not have further testing).

Tolerability was assessed for all infusions administered to patients undergoing prophylaxis and on-demand treatment. Tolerability of 6,497 wilate infusions with an available rating (92.5% of all infusions) was rated as “excellent” (6,254, 96.2%), “satisfactory” (238, 3.7%), or “unsatisfactory” (5, 0.1%) ( Table 2 ). The five infusions with tolerability rated as “unsatisfactory” had been administered to four of the eight patients with an ICSR and were related to hypersensitivity.

**Table 2:** Tolerability assessment of wilate infusions by reason for administration

Reason for administration	Tolerability assessment (patient)			Total number of infusions, *n*
	Excellent, *n* (%)	Satisfactory, *n* (%)	Unsatisfactory, *n* (%)	
Prophylaxis	5,393 (98.1)	97 (1.8)	4 (0.1)	5,494
Bleeding	654 (92.2)	55 (7.8)	0 (0)	709
Surgery	42 (95.4)	1 (2.3)	1 (2.3)	44 b
Menstruation	127 (78.4)	35 (21.6)	0 (0)	162
Prevention a	38 (43.2)	50 (56.8)	0 (0)	88
*Total*	*6,254 (96.2)*	*238 (3.7)*	*5 (0.1)*	*6,497*

Abbreviation: *n* , number of infusions.

Note: For infusions administered for surgeries, only those with available tolerability assessments are presented. Any one wilate infusion may have been administered for more than one reason (e.g., for the treatment of a bleeding episode and menstruation).

a Administered for the purpose of preventing recurrent BEs in patients undergoing on-demand treatment or surgery only.

b Investigator rating.

In 10 of the 47 assessed patients, prothrombin F1 + 2 and/or D-dimer levels increased >2 times the upper limit of normal from preinfusion to postinfusion measurements, but no ICSRs potentially related to thromboembolic events were reported in any of the patients with elevated F1 + 2 and/or D-dimer levels. No thromboembolic events were reported.

### Immunogenicity

In total, 63 patients underwent anti-VWF antibody testing either at baseline or during the course of the study. Those who tested positive for an anti-VWF antibody were then tested for VWF inhibitors. Three patients who were antibody positive, but inhibitor negative at the start of the study, tested positive for VWF inhibitors on at least one occasion. Two of these patients tested negative at subsequent visits after the positive test. One was subsequently found to not have VWD but another, uncharacterized, bleeding disorder, and the other underwent on-demand treatment for approximately 35 months and experienced 20 BEs, all of which were treated successfully. The third patient had VWD type 3 and tested inhibitor positive on two occasions but experienced no clinical symptoms at the time of the positive inhibitor results. She received no further antibody testing; subsequent VWF:RCo recovery testing showed no clinically significant results, and she continued to receive regular prophylaxis with wilate for almost 2 years with “excellent” tolerability and “excellent” or “good” effectiveness.

### Effectiveness in Prophylaxis

The median dose of wilate administered for prophylaxis was 69.3 IU/kg per week (range: 8.2–298.6) in 25 patients (3 type 1 VWD, 8 type 2 VWD, and 14 type 3 VWD). The 25 patients were treated for a mean of 192.1 EDs (SD: 153.8) and a median of 145 EDs (range: 11–512). The mean number of infusions per week was 2 (SD: 1) and the median was 2 (range: 0.5–4.2). Of the 25 patients, 17 received continuous prophylaxis for over 3 months with no treatment gaps exceeding 14 days or for at least 1 year with an average of 1 infusion per week and received a mean of 2.4 (SD: 0.9) infusions per week.

Eighteen of the 25 (72%) patients experienced 233 breakthrough BEs, while 7 (28%) had no bleeding on prophylaxis. Most BEs were mild or moderate in severity (190; 81.5%) and 20 (8.6%) were severe. The majority of the BEs were spontaneous (154 BEs, 66.1%; Table 3 ). The mean ABR was 4.9 (SD: 6.8) and the median was 1.9 (range: 0–27.0) at study end ( Table 4 ). In the 14 children on prophylaxis (11 with type 3 VWD and 3 with type 2 VWD), the mean ABR was 6.1 (SD: 7.3) and the median was 3.0 (range: 0–27.0) at study end.

**Table 3:** Analysis of bleeding characteristics and wilate treatment

Population a	Patients experiencing bleed, *n* (%)	Number of bleeds	Type			Severity				Treatment with wilate			
			Spontaneous, *n* (%)	Trauma, *n* (%)	Other/ unknown, *n* (%)	Mild, *n* (%)	Moderate, *n* (%)	Severe, *n* (%)	Unknown, *n* (%)	1–2 infusions, % ( *n* )	>2 infusions, % ( *n* )	Infusions/treatment, median (range)	Dose [IU/kg], median (range)
Prophylaxis ( *N* = 25)	18 (72.0)	233	154 (66.1)	42 (18.0)	37 (15.9)	87 (37.3)	103 (44.2)	20 (8.6)	23 (9.9)	85.1 (149/175)	14.9 (26/175)	1 (1–14)	55.4 (8.3–1441.3)
On-demand ( *N* = 29)	25 (86.2)	150	102 (68.0)	31 (20.7)	17 (11.3)	27 (18.0)	107 (71.3)	15 (10.0)	0 (0)	94.2 (130/138)	5.8 (8/138)	1 (1–13)	33 (8.3–625)
Menstrual bleeding ( *N* = 9)	9 (100)	56	–	–	–	5 (8.9)	20 (35.7)	26 (46.4)	5 (8.9)	46.4 (26/56)	53.6 (30/56)	3 (1–7)	79.1 (10–339.8)

a Two patients changed from on-demand treatment to prophylaxis, and three patients changed from prophylaxis to on-demand treatment. For all analyses by treatment regimen, these five patients were included in both analysis populations. Seven patients treated on-demand and two treated prophylactically also received treatment for menstrual bleeding.

**Table 4:** Annualized bleeding rates in the prophylaxis population

Type of bleeding event	Prophylaxis ( *N* = 25)	
	Mean (SD)	Median (range)
Spontaneous	3.0 (4.7)	1.5 (0–19.7)
Traumatic	0.7 (1.6)	0 (0–5.7)
Other/ unknown	1.2 (3.6)	0 (0–18.0)
*Total*	*4.9 (6.8)*	*1.9 (0–27.0)*

Abbreviation: SD, standard deviation.

**Fig. 2:**
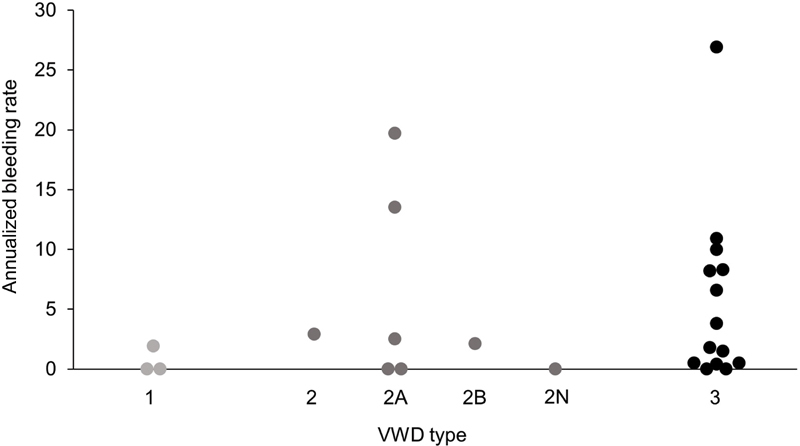
Annualized bleeding rates during wilate prophylaxis by VWD type ( *N* = 25). VWD, von Willebrand disease.

There was a trend for higher ABRs in the patients with VWD type 3 than those with VWD type 2 or 1 ( Fig. 2 ). The three patients with type 1 VWD had experienced severe BEs prior to the study, and two of these patients had previously received prophylaxis with plasma-derived FVIII/VWF.

**Fig. 3:**
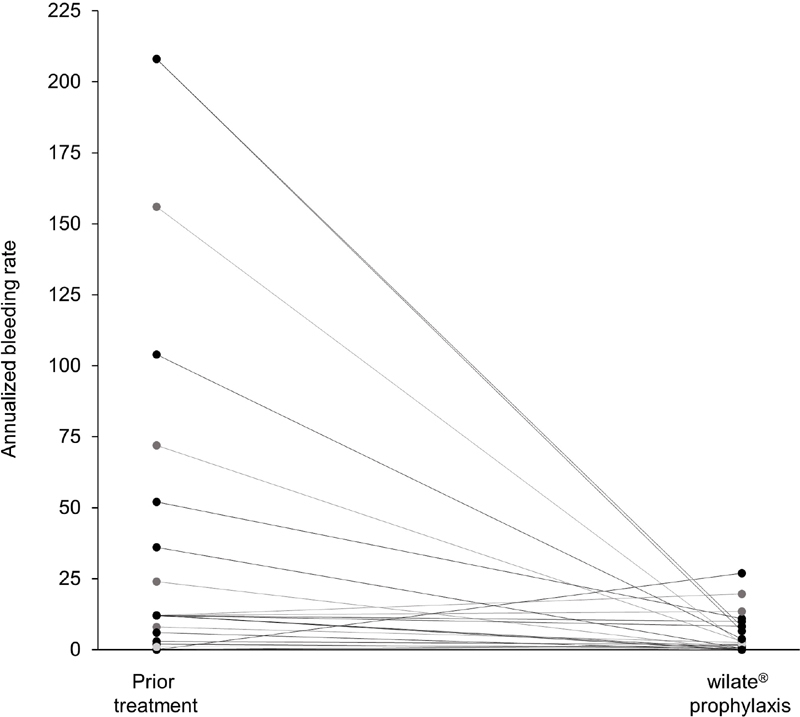
Individual annualized bleeding rates in the 6 months prior to entering the study and during wilate prophylaxis ( *N* = 25).

In the 6 months prior to entering in the study, the patients had a mean ABR of 39.1 (SD: 61.5) and a median ABR of 12 (range: 0–208). Eighteen (72%) patients had received prophylactic treatment, 4 (16%) were treated on-demand, and 3 had no previous treatment (12%) during that period. Comparison of the previous and on-study ABRs in individual patients shows that the majority (76%) had a reduction in bleeding under wilate prophylaxis ( Fig. 3 ).

### Effectiveness in the Treatment of Bleeding

The number, type, severity, and treatment of breakthrough BEs in prophylaxis, in on-demand treatment of BEs, and in treatment of menstrual bleeding are shown in Table 3 . The most common breakthrough bleeds in the patients treated prophylactically were nose bleeds (134 BEs, 58%), bleeds in the gastrointestinal system (44 BEs, 19%), and in the oral cavity (15 BEs, 6%). Forty of the 44 gastrointestinal BEs occurred in a single patient with angiodysplasia. Of the 233 breakthrough BEs, 58 did not require treatment. Most of the treated breakthrough BEs (149/175, 85.1%) required only one to two wilate infusions. The median dose of wilate per breakthrough BE was 55.4 IU/kg (range: 8.3–1,441.3). Treatment of breakthrough BEs was rated successful (“excellent” or “good” effectiveness) by 98% of patients (151 of 154 rated breakthrough BEs) and 99% of investigators (137 of 139) in the prophylactic population. In children, treatment of breakthrough BEs was rated successful (“excellent” or “good” effectiveness) by all patients (101 rated breakthrough BEs) and investigators (110 rated breakthrough BEs) in the prophylactic population.

The most common bleeds in the 29 patients treated on-demand were nose bleeds (65 BEs, 43%) and oral cavity bleeds (23 BEs, 15%). Of the 150 BEs, 11 did not require treatment (treatment status of one BE was unknown). The majority of treated BEs (130/138; 94.2%) required only 1 to 2 wilate infusions; 8 BEs (5.8%) required more than 2 infusions. The median dose of wilate per on-demand BE was 33.0 IU/kg (range: 8.3–625). The on-demand treatment of BEs with wilate was considered “successful” for 100% of 136 BEs rated by the patients and 100% of 127 BEs rated by the investigators.

Nine patients had a total of 56 menstrual BEs treated with wilate, of which 5 (8.9%) were mild, 20 (35.7%) moderate, 26 (46.4%) severe, and 5 (8.9%) of unknown severity. The median treatment duration of menstrual BEs was 3 days (range: 1–7) and the median dose of wilate administered to treat a menstrual BE was 79.1 IU/kg (range: 10–339.8). wilate treatment was “successful” in the treatment of 40 of 48 BEs (83.3%) rated by patients and 26 of 34 BEs (76.5%) rated by the investigator. In none of the treated BEs was a lack of treatment effectiveness reported.

### Effectiveness in Surgery

A total of 62 patients, 8 of whom had VWD type 3, underwent 98 surgical procedures treated with wilate ( Table 1 ). Of the 98 surgeries, 46 (47%) were major (43 [93.5%] planned, 3 [6.5%] emergency) and 52 (53%) were minor (48 [92.3%] planned, 4 [7.7%] emergency). The most frequent major surgical interventions were gynecological and orthopedic procedures. The most common minor interventions were dental procedures.

In minor surgeries, the median number of infusions was 1 (range: 1–19), and the median number of EDs was 1 (range: 1–15). In major surgeries, the median number of infusions was 4 (range: 1–24), and the median number of EDs was 2.5 (range: 1–14). For minor surgery, the mean dose of wilate per procedure was 86.3 IU/kg (SD: 137) and the median dose was 34.2 IU/kg (range: 6.3–679.6). For major surgery, the mean dose per procedure was 144.8 IU/kg (SD: 107.8) and the median dose was 110.8 IU/kg (range: 21.8–500).

Effectiveness assessment was available for 51 of 52 minor surgeries (16 in children and 35 in adults) and for all 46 major surgeries (9 in children and 37 in adults). The effectiveness of wilate was rated as “excellent” in 87 surgeries (90%), “good” in 9 surgeries (9%), and “moderate” in 1 surgery (1%). The one instance in which the effectiveness of wilate was rated as “moderate” occurred in a 9-year-old patient undergoing a minor dental procedure, i.e., a planned tooth extraction. The patient received 1 infusion of 900 IU before the extraction and 3 infusions of 900 IU daily of wilate for 3 days after extraction to control bleeding. The same patient underwent 4 additional dental procedures (3 extractions and 1 gum opening) during the study period, with hemostatic effectiveness rated as “excellent” in 3 and “good” in 1 of these 4 procedures. In the 24 procedures in children, the effectiveness of wilate was rated as “excellent” in 20 surgeries (83.3%), “good” in 3 surgeries (12.5%), and “moderate” in 1 surgery (4.2%).

Concomitant antithrombotic medication was administered in 4 procedures (3 major and 1 minor) of which 3 (2 major and 1 minor) had an effectiveness rating of “excellent” and 1 major procedure had an effectiveness rating of “good.”

## Discussion

This observational, phase 4, postmarketing study collected effectiveness and safety outcome data from patients with all types of VWD treated with wilate in real-world clinical practice. wilate was effective and well tolerated as prophylaxis, for the treatment of acute BEs, and during surgical procedures. The results are in line with data obtained from clinical trials with wilate 6 7 8 9 12 and other studies evaluating wilate in the real-world setting. 13 14 No unexpected ADRs occurred during the observation period.

Prophylaxis in VWD is not as widespread as in hemophilia A, even in patients with VWD type 3 who are most likely to benefit from this treatment modality. There are limited published data on this strategy in VWD patients 4 and this study is, to the best of our knowledge, the first long-term study to examine real-life clinical use of a VWF/FVIII concentrate in prophylaxis. We show that prophylaxis with wilate reduced the number of BEs per year compared with the patients' previous treatment in 76% of patients, with a reduction in mean ABR from 39.1 prior to the study to 4.9. Due to the nature of noninterventional studies, the prophylaxis regimens were not standardized, but the median frequency and dose of wilate prophylaxis in this real-life study were within the recommended range 12 and in line with previous studies with wilate. 6

The effectiveness of treatment of breakthrough bleeds and BEs is similar to pooled results from clinical studies of wilate in which 96% of the 1,095 BEs were successfully treated. 6 The median dose per infusion for the treatment of breakthrough bleeds and BEs in the on-demand population (55.4 and 33 IU/kg, respectively) was within the range of recommendations of the Summary of Product Characteristics for wilate. 12 The treatment of menstrual bleeding was successful for the majority of rated BEs.

Effectiveness in patients undergoing surgery (99% of surgeries were successfully treated) in this study (“excellent” or “good”) is consistent with the findings from a pooled analysis of 57 surgical procedures using wilate (96% rated “excellent” or “good”) 7 and a 96.7% success rate reported in a phase 3 surgery study. 9

Inhibitors to VWF are rare but represent a serious complication of VWD treatment. There is no standard, validated assay that reliably detects clinically relevant neutralizing antibodies to VWF. 15 It is difficult to determine the clinical relevance of experimental VWF antibodies detected by ELISA. In this study, inhibitor development was examined using two experimental assays. None of the three patients with positive VWF inhibitor tests had any clinically significant symptoms, had decreased VWF recovery, or experienced reduced hemostatic effectiveness of wilate. Therefore, there was no clinical evidence of neutralization in the inhibitor-positive patients in this study.

Sustained excessive FVIII:C plasma levels that have been reported for some FVIII-containing VWF products may increase the risk of thrombotic events. 16 17 In this study, there were no thromboembolic complications, consistent with the results from previous studies of wilate treatment that demonstrated a lack of FVIII:C accumulation after repeated dosing. 7 8 9 14 To monitor thromboembolic risk, this study also documented F1 + 2 and D-dimer plasma levels. Due to the noninterventional character of this study, these data were not available for all patients at all time points. Although elevated postinfusion values were documented in a few patients, none were judged to be related to wilate, and no thromboembolic events occurred in any of these patients. Thrombogenicity test parameters are known to be very sensitive to proper blood sampling and handling and to be susceptible to artifactual elevation, so it is likely that the abnormally high values were related to preanalytical issues rather than to coagulation activation.

Evaluating parvovirus B19 seroconversion is difficult, not only because of inconsistent testing, but also because of possible exposure to this pathogen in the community. Due to the production method of wilate, parvovirus B19, if present, would be inactivated; however, empty capsids may remain and nucleic acids can be externalized, which may result in antibody positivity without clinical symptoms. One patient in this study had a symptomatic parvovirus B19 infection, which was unlikely related to treatment with wilate given the known exposure to their child who had acute infectious symptoms at the time.

Owing to the observational nature of the study and blood testing per standard local practice, many tests (e.g., anti-VWF antibody) were not performed in all patients at all time points. These missing data therefore impact the strength of conclusions that can be made in this regard. Another limitation of the study is that most of the patients (80%) were Caucasian, which may not necessarily reflect VWF pharmacokinetics and clinical outcomes across all patients with VWD given the known variation in VWF levels according to race/ethnicity.

Future directions include prospective evaluation of wilate with standardized prophylaxis, validation of an assay for anti-VWF testing, specified inclusion of patients of minority race/ethnicity, and clinical trials to evaluate the efficacy of wilate for excessive menstrual bleeding.

## Conclusion

The results of this observational study confirm that real-world use of wilate is effective and well tolerated for the prevention and treatment of bleeding in patients with all types of VWD. These real-world data further support wilate as an effective treatment option for VWD patients of all types.
