# Supplementary material for: Real-World Data on the Effectiveness and Safety of wilate for the Treatment of von Willebrand Disease
Source: TH Open. 2021 Jul 4;5(3):e264–72. doi: 10.1055/s-0041-1730283 (PMC8255103; doi:10.1055/s-0041-1730283)
Supplement: Supplementary file 1 — Supplementary Material [file 10-1055-s-0041-1730283-s210004.pdf]

Supplementary Table S1 Assessment methods

Tolerability		
Excellent		Very good or good overall feeling during or after the wilate therapy and no ADRs registered
Satisfactory		Moderate overall feeling during or after the wilate therapy and/or occurrence of mild ADRs (e.g., mild headache, mild dizziness, etc.)
Unsatisfactory		Bad overall feeling during or after wilate therapy and/or occurrence of moderate or severe ADRs
Effectiveness in breakthrough bleeds/bleeding events/menstrual bleeds in prophylaxis/on-demand treatment		
Excellent	Bleeding was completely stopped within a reasonable period of time	
Good	Bleeding was completely stopped, but time and/or dose slightly exceeded expectation	
Moderate	Bleeding could be stopped only by significantly exceeding time and/or dose expectation	
None	Bleeding could be stopped only by using other FVIII/VWF-containing products	
Effectiveness in surgical procedures		
Excellent	Hemostasis clinically not significantly different from normal	
Good	Mildly abnormal hemostasis in terms of quantity and/or quality (e.g., slight oozing)	
Moderate	Moderately abnormal hemostasis in terms of quantity and/or quality (e.g., moderate, controllable bleeding)	
None	Severely abnormal hemostasis in terms of quantity and/or quality (e.g., severe hemorrhage that is difficult to control)	

Abbreviations: ADR, adverse drug reaction; FVIII, factor VIII; VWF, von Willebrand factor.

 © 2021. The Author(s).

Supplementary Table S2 Baseline VWF and FVIII levels (local laboratory measurements)

**Baseline values, VWF subtype**	**All patients ($N = 111$)[a]**
Baseline VWF:RCo (%), mean (SD)	
1 ($n = 47$)	36.5 (21.6)
2, not specified ($n = 5$)	24.5 (10.5)
2A ($n = 12$)	13.1 (14.9)
2B ($n = 5$)	27.4 (9.8)
2M ($n = 3$)	3.4 (5.7)
2N ($n = 4$)	57.1 (32.3)
3 ($n = 16$)	8.2 (14.9)
Unknown ($n = 3$)	13.3 (14.7)
Not applicable[b] ($n = 1$)	123
Total ($n = 96$)	27.8 (24.9)
Baseline FVIII:C (%), mean (SD)	
1 ($n = 45$)	54.3 (29.3)
2, not specified ($n = 5$)	44.4 (17.1)
2A ($n = 11$)	44.0 (23.9)
2B ($n = 5$)	54.2 (8.4)
2M ($n = 3$)	10.8 (18.4)
2N ($n = 4$)	28.9 (23.4)
3 ($n = 18$)	8.2 (11.0)
Unknown ($n = 7$)	43.6 (4.5)
Not applicable[b] ($n = 1$)	49.4
Total ($n = 99$)	41.1 (28.8)

Abbreviations: FVIII, factor VIII; SD, standard deviation; VWF, von Willebrand factor.
[a]VWF data were missing for 15 patients and FVIII data were missing for 10 patients.
[b]One patient was diagnosed during the study as having hemophilia A rather than VWD.

 © 2021. The Author(s).
